# Clinical risk factors for postoperative infection in adult cardiac surgery with cardiopulmonary bypass: a retrospective study

**DOI:** 10.1016/j.infpip.2025.100458

**Published:** 2025-04-08

**Authors:** Guangxu Mao, Wensen Chen, Liyun Wang, Sheng Zhao, Feng Zang

**Affiliations:** a Department of Infection Management, Xinghua People's Hospital Affiliated to Yangzhou University, Xinghua, Jiangsu, China; b Department of Infection Management, The First Affiliated Hospital with Nanjing Medical University, Nanjing, Jiangsu, China; c Department of Cardiovascular Surgery, The First Affiliated Hospital with Nanjing Medical University, Nanjing, Jiangsu, China

**Keywords:** Cardiac surgery, Cardiopulmonary bypass, Postoperative infection, Nomogram, Risk factors

## Abstract

**Background:**

Postoperative infection remains a serious problem for patients undergoing open-heart surgery and is associated with poor prognosis and mortality.

**Aim:**

To determine the incidence, characteristics and associated risk factors for nosocomial infections in adult cardiac surgery patients and to develop a nomogram prediction model.

**Methods:**

Data were retrospectively collected from patients who underwent cardiac surgery with cardiopulmonary bypass (CPB) at a tertiary hospital in 2023. Patients were divided into an infected group (*N* = 130) and a non-infected group (*N* = 192). Multivariate logistic regression analysis was used to analyse the independent risk factors for healthcare-associated infections after cardiac surgery under CPB.

**Results:**

Of the 1584 patients, 130 (8.21%) developed postoperative infections (infection group). Lower respiratory tract was the most common site of infection (*N* = 74, 56.9%), while Gram-negative bacteria were the predominant isolates overall (*N* = 81, 62.3%). Among the Gram-negative bacteria, *Acinetobacter baumannii* was the most frequently identified, whereas *Staphylococcus aureus* was the leading strain among Gram-positive bacteria. Multivariate logistic regression analysis of the 322 patients included in the study revealed that CPB duration, American Society of Anaesthesiologists score, procalcitonin concentration on the first postoperative day, monocyte:lymphocyte ratio, preinfection mechanical ventilation duration, and preinfection central venous catheterization duration were the six independent predictors of postoperative infection. The area under the receiver operating characteristic curve was 0.824 (0.778–0.870), and the model showed good predictive performance.

**Conclusion:**

A nomogram has been developed to predict postoperative infection via commonly available data. This tool could assist clinicians in optimising the perioperative care of patients undergoing cardiac surgery with CPB, but further external validation is needed.

## Introduction

Cardiopulmonary bypass (CPB) technology represents a pivotal auxiliary tool in cardiovascular and vascular surgery, having markedly advanced these fields since its inception [1]. This technology provides a transparent, bloodless surgical field by temporarily substituting the functions of the heart and lungs, thereby facilitating intricate heart surgeries. Cardiac surgeries utilizing CPB represent a significant proportion of all cardiovascular surgeries. According to data from the Chinese Society of Biomedical Engineering, Extracorporeal Circulation Branch, a total of 263,292 cardiovascular surgeries were conducted in China in 2022, with 159,949 of the surgeries employing CPB, representing 60.7% of the surgeries. Despite the marked improvement in survival rates and quality of life due to cardiac surgery employing CPB technology, its associated complications, particularly postoperative infections, remain critical factors affecting patient outcomes. Postoperative infections are common following cardiac surgery, and the most frequently reported infections are pneumonia, surgical site infection (SSI), mediastinitis, and bacteraemia [2]. A comprehensive review of the literature from national and global sources revealed considerable variation in the incidence of infections after cardiac surgery, with rates ranging from 4.2% to 26.2% [[3], [4], [5]]. Additionally, a substantial retrospective study conducted in China revealed that the prevalence of healthcare-associated infections (HAIs) among patients undergoing cardiac and vascular surgery reached as high as 8% [6]. Postoperative infection not only prolongs hospital stays and increases medical costs, but also may lead to surgical failure and pose significant risks to patients' lives; therefore, identifying and analysing the risk factors for postoperative infection in adult cardiac surgery involving CPB is very important. This study aimed to identify potential risks associated with postoperative infections, provide a scientific foundation for developing targeted prevention strategies, reduce the incidence of postoperative infection, enhance patient outcomes, and improve the overall efficacy of cardiovascular surgeries.

## Methods

### Study subjects

This research employed a matched case–control methodology, focusing on individuals who underwent cardiac and vascular surgery involving CPB at a tertiary hospital between January and December 2023. A total of 1584 cases were included in the analysis. Patients were matched with controls at a ratio of 1:1.5, considering factors such as age, sex, underlying diseases, and type of surgery. Ultimately, 322 patients were successfully enrolled, comprising 130 patients with infections and 192 without infections. Ethical approval for this study was obtained from the Ethics Committee (2024-SR-535).

### Case definition

#### Diagnostic criteria

The primary objective of this research was to determine the prevalence of new HAIs following cardiac surgery. The diagnostic criteria were aligned with the surveillance definitions set forth by the Centers for Disease Control and Prevention (CDC) in the USA and the National Healthcare Safety Network (NHSN) [7]. Postoperative infections are diagnosed by infectious disease specialists, microbiology laboratories and hospital infection control staff.

#### Inclusion criteria

The inclusion criteria were as follows: (1) patients who underwent cardiac surgery with CPB; (2) patients who were aged ≥18 years; (3) patients whose surgical team, comprising surgeons, CPB technicians, and anaesthesiologists, consistently provided care.

#### Exclusion criteria

The exclusion criteria were as follows: (1) patients who had preoperative hospital infections or a temperature of ≥38°C; (2) patients who underwent emergency cardiovascular surgery due to trauma, infective endocarditis, or malignancy; (3) patients who had undergone a second cardiac surgery; (4) patients who were transferred to another hospital or lost to follow-up; (5) patients whose clinical data were incomplete.

### Research methods

#### General information

The clinical data collected included the age, sex, height, weight, and medical history of the patients. The intraoperative dataset included information on the type of surgery, CPB time, duration of surgery, ASA score, National Nosocomial Infections Surveillance (NNIS) score, and number of intraoperative blood transfusions. Furthermore, the preoperative laboratory test data recorded included serum albumin, total cholesterol, monocyte, absolute neutrophil count, total lymphocyte count, platelet count, and haemoglobin levels.

#### Preoperative nutrition and inflammation indicators

Inflammatory and nutritional markers were assessed using the patients' initial blood test results upon admission [8]. The calculation formulas and evaluation criteria are shown in Table I, Table II.

**Table I:** Inflammatory and nutritional markers used in the study

Laboratory indicator	Calculation method
Inflammatory markers:	
Monocyte:lymphocyte ratio	Total no. of monocytes/total no. of lymphocytes
Neutrophil:lymphocyte ratio	Total no. of neutrophil/total no. of lymphocytes
Platelets:lymphocyte ratio	Total no. of platelets/total no. of lymphocytes
Systemic Inflammatory Response Index	Monocyte count × neutrophil count/lymphocyte count

**Table II:** Nutritional markers used in the study

Nutritional markers	CONUT score: ALB + TLC + TC			
	Normal	Mild	Moderate	Severe
Albumin (g/L)	≥35	30–34.9	25–29.9	<25
Score	0	2	4	6
Total lymphocyte count (μL)	≥1600	1200–1590	800–1199	<800
Score	0	1	2	3
Total cholesterol (mmol/L)	≥4.68	3.64–4.67	2.60–3.63	<2.60
Score	0	1	2	3
Total score	0–1	2–4	5–8	9–12

Controlling Nutritional Status (CONUT) score; ALB, albumin; TLC, total lymphocyte count; TC, total cholesterol.

### Statistical methods

Statistical evaluations were conducted using Statistical Package for the Social Sciences (SPSS, version 26.0) and R (version 4.0.2). Data exhibiting a normal distribution and homogeneity of variance are reported as the means ± standard deviations. Independent sample *t*-tests were employed to facilitate comparisons between the two groups. Frequency and percentage data are presented for count data, with comparisons made via the χ^2^-test. A logistic regression model was constructed to identify risk factors for infection following adult cardiac surgery, and the diagnostic efficacy of each indicator was evaluated via receiver operating characteristic (ROC) curves. All the statistical analyses were conducted with two-sided tests, and *P* < 0.05 was considered statistically significant.

## Results

### Incidence and distribution of postoperative infection

In 2023, 1584 patients who met the inclusion criteria for this study underwent cardiac and vascular surgery with CPB. Among the total number of patients, 130 cases of postoperative infection were identified, resulting in an incidence rate of 8.21%. The lower respiratory tract was the most common site of infection (*N* = 74, 56.9%), while Gram-negative bacteria were the predominant isolates overall (*N* = 81, 62.3%). Among Gram-negative bacteria, *Acinetobacter baumannii* was the most frequently identified, whereas *Staphylococcus aureus* was the leading strain among Gram-positive bacteria. Among the total number of postoperative infections, 36 cases (27.6%) were attributed to hospital-acquired infections caused by multidrug-resistant (MDR) bacteria. Table III provides a comprehensive overview of the micro-organisms responsible for the initial postoperative infection and the incidence rates for each postoperative infection type.

**Table III:** Distribution of initial postoperative infection and pathogenic micro-organisms

Pathogenic micro-organisms	LRTI	VAP	BSI	Other	Overall	
					Overall	Of which: MDR
No. of cases	74 (56.92%)	27 (20.77%)	14 (10.77%)	15 (11.54%)	130	36 (27.69%)
Infection incidence rate^a^	4.67	1.7	0.88	0.94	8.21	2.27
Gram negative^b^	49 (66.21%)	18 (66.65%)	9 (64.28%)	5 (33.33%)	81 (62.31%)	
*Acinetobacter baumannii*	12 (16.22%)	7 (25.92%)	3 (21.43%)	2 (13.33%)	24 (18.46%)	18 (50%%)
*Klebsiella pneumoniae*	11 (14.86%)	1 (3.70%)	1 (7.14%)	1 (6.67%)	14 (10.77%)	1 (2.78%)
*Stenotrophomonas maltophilia*	8 (10.81%)	2 (7.41%)	0	0	10 (7.69%)	NA
*Serratia marcescens*	8 (10.81%)	4 (14.81%)	0	0	12 (9.23%)	NA
*Pseudomonas aeruginosa*	7 (9.46%)	4 (14.81%)	1 (7.14%)	2 (13.33%)	14 (10.77%)	5 (13.89%)
Other	3 (4.05%)	0	4 (28.57%)	0	7 (5.38%)	NA
Gram positive^b^	7 (9.46%)	4 (14.81%)	4 (28.56%)	6 (40.00%)	21 (16.15%)	
*Enterococcus* spp.	0	0	2 (14.28%)	0	2 (1.54%)	NA
*Staphylococcus aureus*	7 (9.46%)	3 (11.11%)	0	4 (26.67%)	14 (10.77%)	11 (30.55%)
Coagulase-negative staphylococci	0	1 (3.70%)	1 (7.14%)	2 (13.33%)	4 (3.07%)	1 (2.78%)
Other	0	0	1 (7.14%)	0	1 (0.77%)	NA
Fungal^b^	7 (9.46%)	3 (11.11%)	1 (7.14%)	0	11 (8.46%)	
*Aspergillus* spp.	0	1 (3.70%)	0	0	1 (0.77%)	NA
*Candida* spp.	7 (9.46%)	2 (7.41%)	1 (7.14%)	0	10 (7.69%)	NA

LRTI, lower respiratory tract infection; VAP, ventilator-associated pneumonia; BSI, bloodstream infection; NA, not applicable; MDR, multidrug-resistant.

Data are presented as absolute frequency (% of the subgroup).

Strains have not been isolated from some infected patients.

a Infection incidence rate, denominator: 1584.

b Constituent ratio, denominator: no. of patients per site of infection.

### Analysis of risk factors for postoperative infections following cardiovascular surgery with CPB

**Figure 1:**
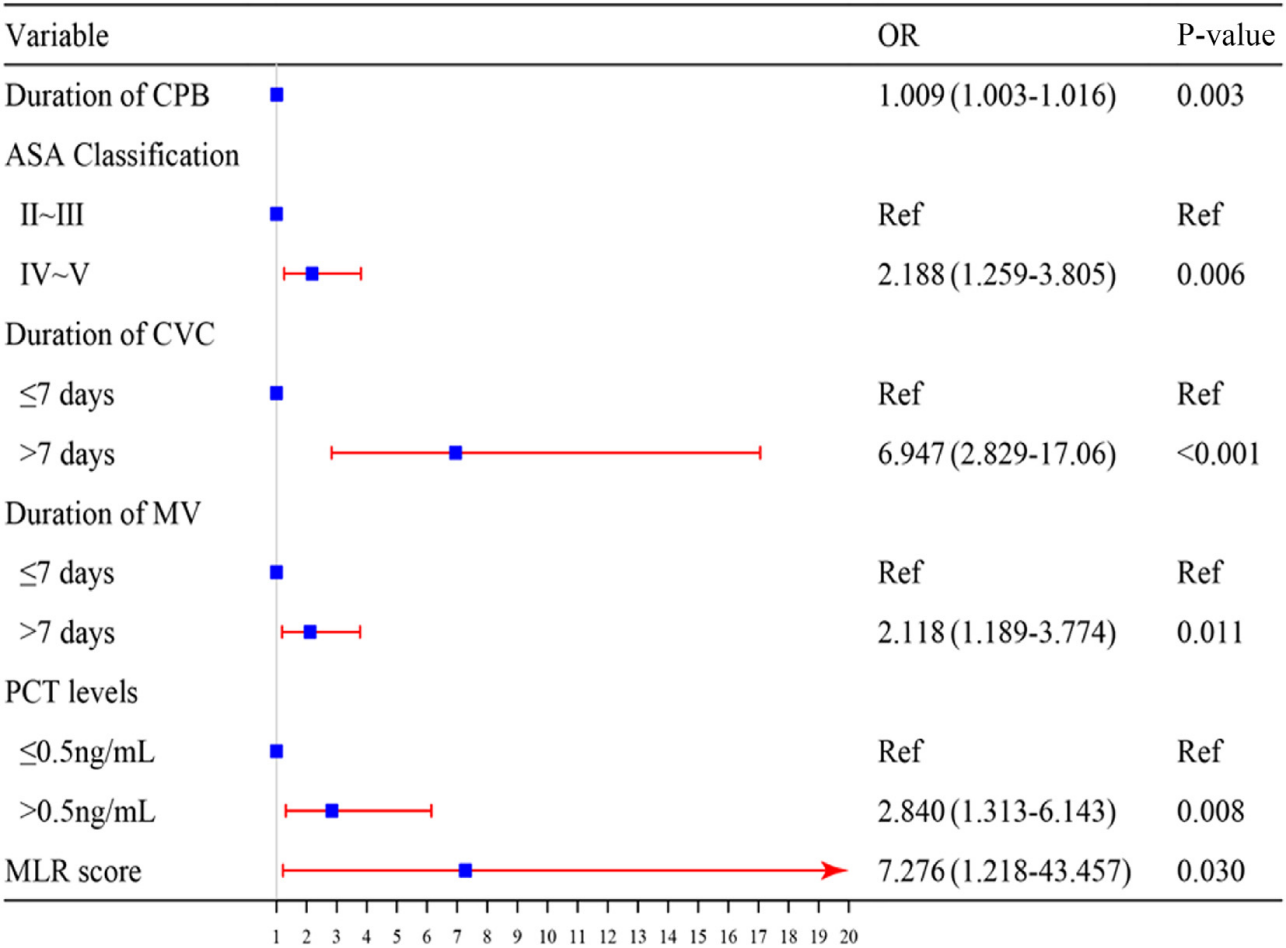
Forest plot of risk factors for postoperative infections following cardiopulmonary bypass (CPB) and major cardiovascular surgery. OR, odds ratio; ASA, American Anesthesiologists Association; CVC, central venous catheterization; MV, mechanical ventilation; PCT, procalcitonin; MLR, monocyte:lymphocyte ratio.

**Figure 2:**
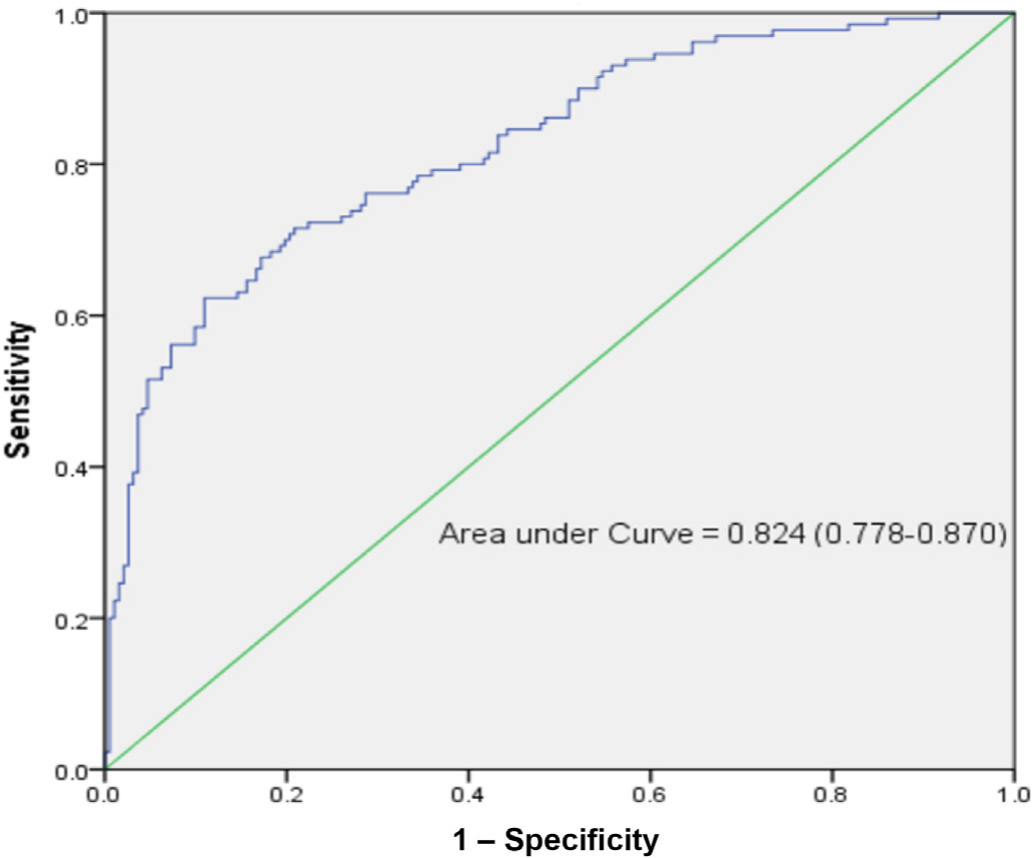
Receiver operating characteristics curve of the nomogram prediction model.

The univariate analysis revealed that various factors were significant risk factors for postoperative infections. These included age, diabetes status, hypertension status, NLR, MLR, the systemic inflammatory response index (SIRI), the CONUT score, the duration of CPB, the myocardial block time, the ASA score, the NNIS score, procalcitonin (PCT) concentration on the first postoperative day, chest drainage, nasogastric feeding, the preinfection MV duration, and the preinfection CVC duration. The results of this analysis are detailed in Table IV. These indicators were subsequently included in a logistic multivariate regression analysis, identifying six independent predictors of postoperative nosocomial infections. The independent predictors of postoperative nosocomial infections were the MLR score, CPB duration, ASA score, PCT concentration on the first postoperative day, preinfection MV duration, and CVC duration. A forest plot was constructed based on the findings of the logistic multivariate regression analysis (Figure 1), and the Hosmer–Lemeshow goodness-of-fit test indicated an appropriate fit (χ^2^ = 6.162, *P* > 0.05), suggesting that the model has satisfactory predictive performance. Moreover, the ROC-AUC was 0.824 (95% confidence interval (CI): 0.778–0.870), indicating that the model exhibited robust discriminatory capacity (Figure 2).

**Table IV:** Univariate analysis of postoperative infections

Variable	Infected group (*N* = 130)	Non-infected group (*N* = 192)	*t*/χ^2^	*P-*value
Baseline				
Sex			3.002	0.083
Male	85 (65.38%)	107 (55.73%)		
Female	45 (34.62%)	85 (44.27%)		
Age (years)	62.76 ± 12.63	59.28 ± 12.33	–2.460	0.014
Body mass index (kg/m^2^)	23.40 ± 3.56	24.20 ± 3.77	1.917	0.057
Comorbidities				
Diabetes	25 (19.23%)	20 (10.42%)	5.009	0.025
Hypertension	51 (39.23%)	54 (28.12%)	4.351	0.037
Pulmonary hypertension	21 (16.15%)	18 (9.37%)	3.346	0.067
Cerebrovascular disease	19 (14.61%)	20 (10.42%)	1.284	0.257
Preoperative index				
Platelet:lymphocyte ratio	120.59 ± 52.66	110.07 ± 51.29	–1.787	0.075
Neutrophil:lymphocyte ratio	2.84 ± 2.23	2.19 ± 1.26	–2.978	0.003
Monocyte:lymphocyte ratio	0.38 ± 0.25	0.29 ± 0.12	–3.708	<0.001
SIRI	1.60 ± 2.19	1.05 ± 0.78	–2.742	0.007
CONUT			12.944	0.005
Normal	45 (34.61%)	97 (50.52%)		
Mild	67 (51.54%)	86 (44.79%)		
Moderate	16 (12.31%)	8 (4.17%)		
Severe	2 (1.54%)	1 (0.52%)		
Intraoperative information				
Operation			6.510	0.089
Coronary artery	26 (20%)	20 (10.42%)		
Cardiac valves	81 (62.31%)	127 (66.14%)		
Aorta	23 (17.69%)	45 (23.44%)		
Other	15 (11.54%)	25 (13.02%)		
Duration of CPB (h)	158.45 ± 66.09	122.49 ± 38.75	–5.588	<0.001
Myocardial block time (h)	105.37 ± 44.21	82.76 ± 33.48	–4.989	<0.001
ASA score				
Ⅱ∼Ⅲ	51 (39.23%)	121 (63.02%)	17.631	<0.001
Ⅳ∼Ⅴ	79 (60.77%)	71 (36.98%)		
NNIS score				
0∼1	23 (17.69%)	60 (31.25%)	7.447	0.006
2∼3	107 (82.31%)	132 (68.75%)		
Blood transfusion (≥400 mL)	58 (44.61)	103 (53.64%)	2.529	0.112
Postoperative information				
Procalcitonin (>0.5 ng/mL)	119 (91.54)	134 (69.79%)	21.773	<0.001
Chest drainage	98 (75.38)	166 (86.46%)	6.437	0.011
Nasogastric feeding	66 (50.77)	43 (22.39%)	27.869	<0.001
Preinfection MV duration (>7 days)	50 (38.46)	7 (3.64%)	64.496	<0.001
Preinfection CVC duration (>7 days)	100 (76.92)	82 (42.71%)	36.926	<0.001

SIRI, Systemic Inflammatory Response Index; CONUT, Controlling Nutritional Status; CBP, cardiopulmonary bypass; ASA, American Society of Anaesthesiologists; NNIS, National Nosocomial Infections Surveillance system; MV, mechanical ventilation; CVC, central venous catheterization.

### Column chart of postoperative hospital infection risk

**Figure 3:**
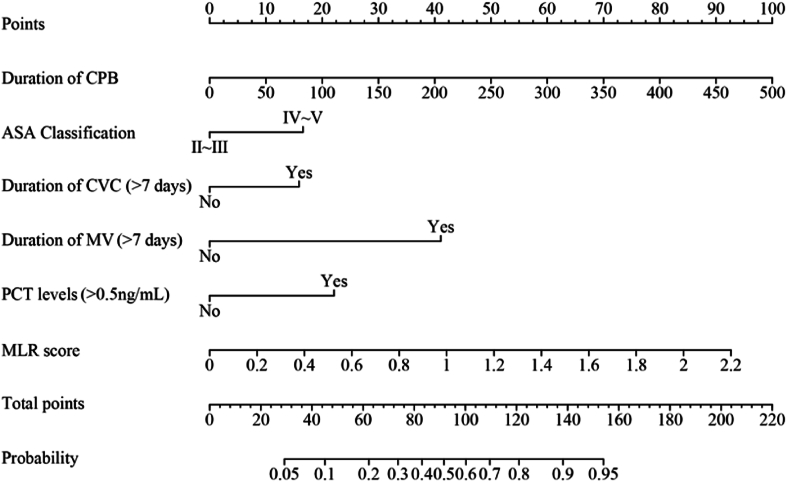
Nomogram model for risk prediction of postoperative infection. CPB, cardiopulmonary bypass and major cardiovascular surgery; ASA, American Anesthesiologists Association; CVC, central venous catheterization; MV, mechanical ventilation; PCT, procalcitonin; MLR, monocyte:lymphocyte ratio.

**Figure 4:**
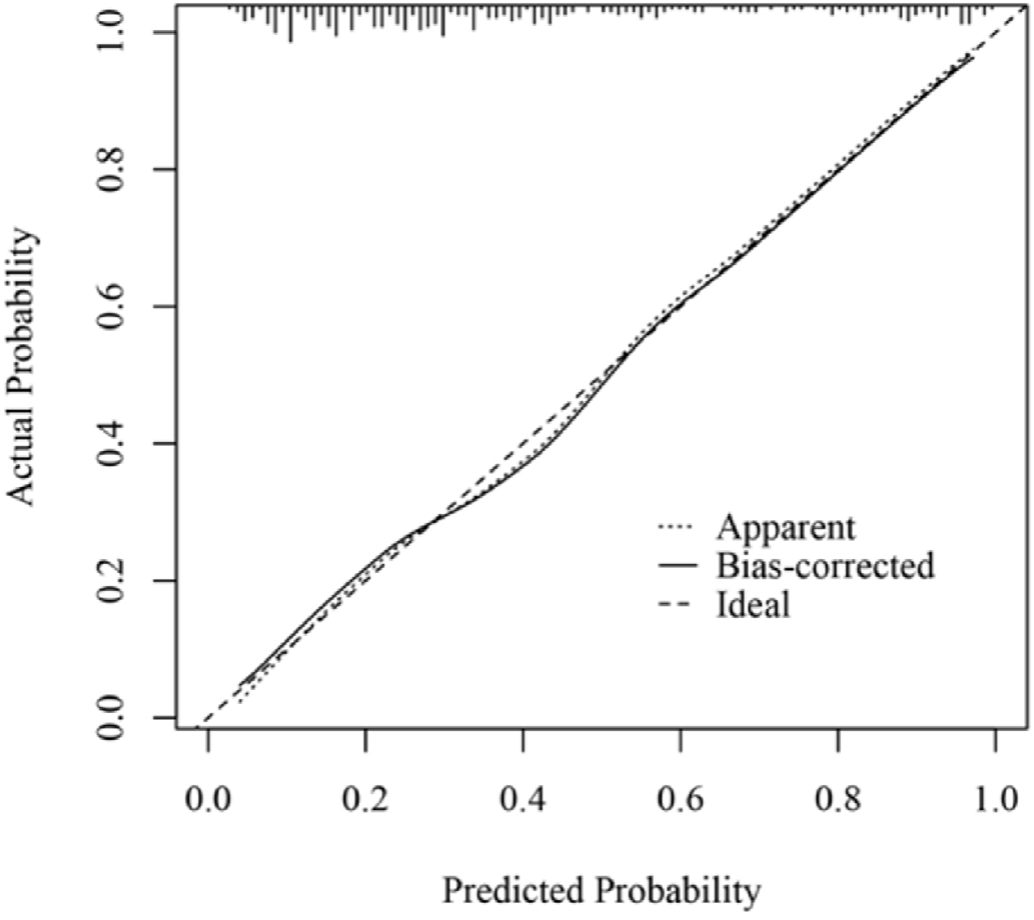
Calibration curve of the prediction model.

**Figure 5:**
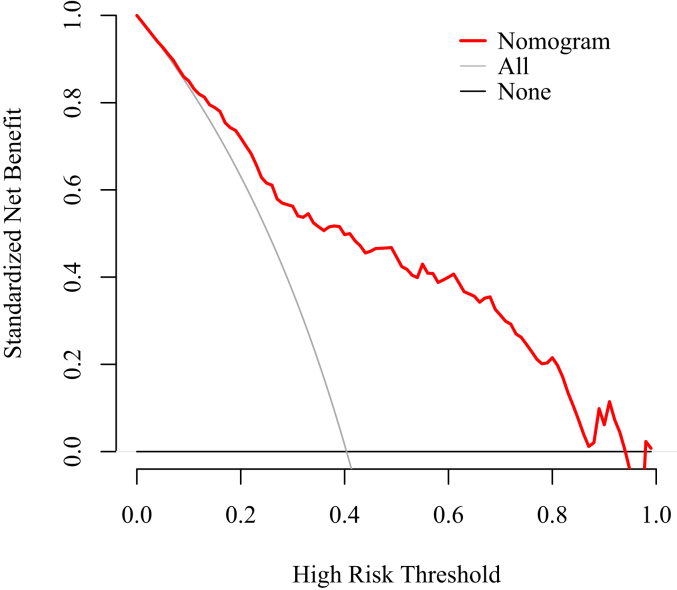
Decision curve analysis of the prediction model.

A predictive model for the risk of postoperative infections was developed through binary logistic multifactorial regression analysis. The column chart in Figure 3 illustrates the practical application of the regression equation, focusing on the duration of the CPB as the most significant contributor, followed by the MLR score. The mean squared error (MSE) was calculated to be 0.00024, whereas the 0.9 quantile of absolute error was 0.029. The calibration curve for the model demonstrated a high degree of alignment with the reference line, indicating strong concordance between the predicted and actual probabilities (Figure 4). Furthermore, the decision curve analysis (DCA) results showed a threshold probability range of 0.03 to 0.94 (Figure 5).

### Impact of postoperative infection on clinical outcomes

**Figure 6:**
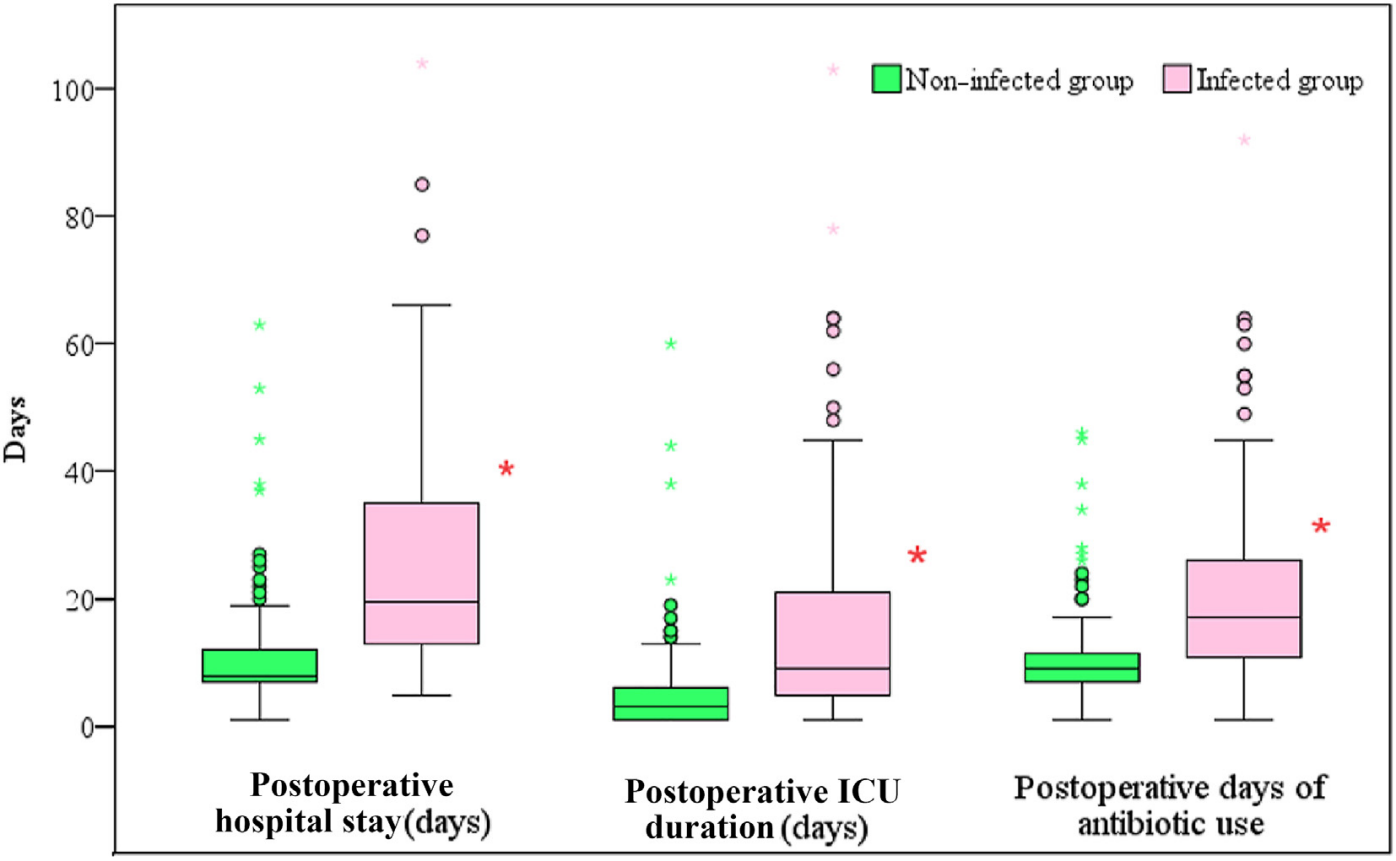
Comparison of clinical outcomes between two groups. ∗Compared with infected patients, *P* < 0.001. ICU, intensive care unit.

Compared with the non-infected group, the infected group exhibited significantly longer postoperative hospitalization, a more extended ICU stay, and an increased duration of antimicrobial drug use (*P* < 0.001) (Figure 6).

## Discussion

Despite recent advances in medical technology and improvements in the operating room environment, the rate of HAIs following heart surgery remains remarkably high. This elevated incidence may be attributed to the surgical procedure's complexity, the CPB's immunosuppressive effects, and the patients' underlying health conditions. In this study, the incidence of postoperative infection was 8.21% (130/1584) following cardiac surgery performed under CPB, which is relatively low compared to international data [9,10]. For particular demographic groups, the postoperative infection rate observed in this study was lower than that reported for infants [11] but higher than the rates identified in children aged < 18 years [12,13] and in elderly individuals (aged ≥70 years) [14]. The most prevalent postoperative infections identified were lower respiratory tract infections (74, 56.92%), ventilator-associated pneumonia (27, 20.77%), and bloodstream infections (14, 10.77%). The extant literature suggests that these infections are common in the postoperative setting, whereas SSIs are comparatively rare [15,16].

A total of 113 micro-organisms were isolated from 130 patients with HAI. The predominant pathogens were Gram-negative bacteria, accounting for 62.31% (81/130) of the isolates. The most common species was *Acinetobacter baumannii*, accounting for 29.63% (24/81) of the Gram-negative isolates. *Staphylococcus aureus* was the most prevalent Gram-positive bacterium, accounting for 66.67% (14/21) of the Gram-positive infections. These findings are consistent with those reported by Renmin Hospital of Wuhan University (Hubei Provincial People's Hospital) in China [6]. A ten-year study on surgical wound infections revealed that the primary pathogens isolated were *Staphylococcus epidermidis* (44.5%) and *Staphylococcus aureus* (31.5%) [17]. Infections caused by *Staphylococcus aureus* often result from inadequate adherence to standard preoperative infection control measures and are associated with a high prevalence of staff carrying primary outbreak strains [18]. Despite the low incidence of SSIs in our hospital, maintaining a concentrated effort in preoperative infection control measures remains essential. The anti-infective treatment of Gram-negative bacteria should be the primary objective following CPB, with clinical interventions guided by drug sensitivity testing results. The current perioperative prevention guidelines recommend the use of first- and second-generation cephalosporins, which have been demonstrated to exhibit limited efficacy against certain Gram-negative bacteria. MDR strains accounted for 27.69% of the isolated strains, with an infection incidence of 2.27%; most patients presented with multiple bacterial infections. While the prevalence of MDR infections varies across regions, hospitals, and wards, the overall burden remains comparable [10]. The considerable difficulties that bacterial resistance presents for clinical anti-infection treatments are a significant challenge. Consequently, cardiac surgeons must maintain a high level of vigilance to mitigate the risk of infection.

In this study, we developed a simple model that employs preoperative and intraoperative clinical variables, in conjunction with biomarkers, to predict the likelihood of infection following open heart surgery conducted under CPB. A line chart was constructed to demonstrate these projections. In line with prior research, our findings substantiate that the preinfection MV duration, preinfection CVC duration, and ASA score are independent risk factors for postoperative infections. Moreover, the duration of CPB has been identified as a significant independent risk factor [2,19]. The suppression of the immune system during CPB may reduce the patient's ability to combat pathogens. Moreover, in the absence of adequate disinfection, equipment employed in CPB, including tubing and oxygenators, may act as conduits for bacterial invasion. A study comprising 6500 patients who underwent open-heart surgery with CPB revealed that a perfusion time >120 min was associated with an OR of 2.45 (95% CI: 1.63 to 3.67) for infections. Similarly, an aortic cross-clamping time >120 min was associated with an OR of 2.31 (1.34 to 3.98); in contrast, an intervention time >300 min was linked to an OR of 2.78 (1.47 to 5.28), both of which were related to primary bloodstream infections (BSIs) [20]. This study employs an innovative composite index of nutrition and inflammation – including the NLR, MLR, SIRI, PLR, and COUNT – in a manner that has rarely been employed in prior research on postoperative complications following cardiac surgery. In contrast to single markers, these composite indicators offer a more comprehensive evaluation of a patient's inflammatory and nutritional status, thereby improving the prediction of clinical outcomes across a range of diseases [21]. These results indicate that the MLR is an independent risk factor for postoperative infection. Several retrospective studies corroborate this finding. An elevated MLR is associated with an increased risk of poor outcomes and infection following cardiac intervention. The MLR is a marker of potential infection risk and a tool to inform clinical decision-making, enhancing monitoring and prevention strategies [22,23]. Furthermore, PCT testing conducted within 48 h post-surgery has been demonstrated to be an effective predictor of postoperative infection, exhibiting diagnostic accuracy that is considered to be superior to that of C-reactive protein (CRP) [24,25]. The guidelines emphasize that the early identification and appropriate treatment of infections are vital for improving patient outcomes [26]. In this study, the selection of indicators focused on data collected preoperatively and intraoperatively, with postoperative indicators limited to PCT concentrations measured on the first day following surgery. Our preliminary yet unpublished research findings suggest that the diagnostic significance of absolute postoperative PCT levels exceeds that of PCT variation rates and white blood cell (WBC) counts, thereby highlighting their potential as more informative biomarkers. However, some studies indicate that the widespread use of extracorporeal circulation in cardiac surgery may induce an acute inflammatory response, which could reduce the diagnostic accuracy of biomarkers for bacterial infections [25,27].

It is essential to acknowledge the limitations of this study, which may influence the interpretation of the findings. First, the retrospective, single-centre design limits the generalizability of the findings due to the restricted sample size. Consequently, the results are not definitive, and further multicentre studies are needed to validate the findings and provide more robust data. Second, the limited sample size constrained the survey to include only patients who underwent cardiac surgery without subclassifying them into specific conditions such as congenital malformations, coronary artery bypass grafting, or valve replacement.

## Conclusions

The six independent predictors of postoperative infection were CPB duration, ASA score, PCT concentration on the first postoperative day, MLR score, preinfection MV duration, and CVC duration. This study comprehensively examines the incidence and types of postoperative infections, bacterial detection, and adverse outcomes following cardiac surgery. The developed predictive model effectively facilitates the early identification of postoperative infections in this context. This capability permits the prompt implementation of intervention strategies for high-risk patients, thereby facilitating the formulation of more precise treatment and monitoring plans to prevent complications. In the future, an integrated clinical decision support system based on this risk prediction model will provide real-time data analysis and risk assessment, enhancing clinical decision-making with robust informational support and reducing reliance on subjective judgement.

## Author contributions

Feng Zang conceived and designed the study, and secured the funding. Guangxu Mao wrote the manuscript. Other authors were involved in the investigation and validation of data. Feng Zang was involved in study methodology and managed the statistical analysis.

## Funding

This study was supported by Young Scholars Fostering Fund of the First Affiliated Hospital of Nanjing Medical University (PY202434) and the Research Project on Hospital Management Innovation of Jiangsu Hospital Association (JSYGY-3-2020-756).

## Conflict of interest statement

None declared.

## References

[1] Baehner T., Boehm O., Probst C., Poetzsch B., Hoeft A., Baumgarten G. (2012). Kardiopulmonaler bypass in der Herzchirurgie. Anaesthesist.

[2] Abukhodair A., Alqarni M.S., Alzahrani A., Bukhari Z.M., Kadi A., Baabbad F.M. (2023). Risk factors for postoperative infections in cardiac surgery patients: a retrospective study. Cureus.

[3] Massart N., Mansour A., Ross J.T., Piau C., Verhoye J.P., Tattevin P. (2022). Mortality due to hospital-acquired infection after cardiac surgery. J Thorac Cardiovasc Surg.

[4] de la Varga-Martínez O., Gómez-Sánchez E., Muñoz M.F., Lorenzo M., Gómez-Pesquera E., Poves-Álvarez R. (2021). Impact of nosocomial infections on patient mortality following cardiac surgery. J Clin Anesth.

[5] Kollef M.H., Sharpless L., Vlasnik J., Pasque C., Murphy D., Fraser V.J. (1997). The impact of nosocomial infections on patient outcomes following cardiac surgery. Chest.

[6] Jiang W., Hu X., Hu Z., Tang Z., Wu H., Chen L. (2018). Morbidity and mortality of nosocomial infection after cardiovascular surgery: a report of 1606 cases. Curr Med Sci.

[7] Horan T.C., Andrus M., Dudeck M.A. (2008). CDC/NHSN surveillance definition of health care-associated infection and criteria for specific types of infections in the acute care setting. Am J Infect Control.

[8] Galizia G., Lieto E., Auricchio A., Cardella F., Mabilia A., Podzemny V. (2017). Naples Prognostic Score, based on nutritional and inflammatory status, is an independent predictor of long-term outcome in patients undergoing surgery for colorectal cancer. Dis Colon Rectum.

[9] Liu Z., Zhang X., Zhai Q. (2021). Clinical investigation of nosocomial infections in adult patients after cardiac surgery. Medicine.

[10] Ren J., Duan S., Wu Y., Wen M., Zhang J., Liu Y. (2023). Multidrug-resistant bacterial infection in adult patients following cardiac surgery: clinical characteristics and risk factors. BMC Cardiovasc Disord.

[11] Levy I., Ovadia B., Erez E., Rinat S., Ashkenazi S., Birk E. (2003). Nosocomial infections after cardiac surgery in infants and children: incidence and risk factors. J Hosp Infect.

[12] Tweddell S., Loomba R.S., Cooper D.S., Benscoter A.L. (2019). Health care-associated infections are associated with increased length of stay and cost but not mortality in children undergoing cardiac surgery. Congenit Heart Dis.

[13] Turcotte R.F., Brozovich A., Corda R., Demmer R.T., Biagas K.V., Mangino D. (2014). Health care-associated infections in children after cardiac surgery. Pediatr Cardiol.

[14] Gao Y., Wang C., Wang Y., Li J., Wang J., Wang S. (2022). Establishment and validation of a nomogram to predict hospital-acquired infection in elderly patients after cardiac surgery. Clin Interv Aging.

[15] Horvath K.A., Acker M.A., Chang H., Bagiella E., Smith P.K., Iribarne A. (2013). Blood transfusion and infection after cardiac surgery. Ann Thorac Surg.

[16] Talwar S., Airan B., Singh S.P., Sahu M.K., Siddharth C.H.B., Devagouru V. (2017). Hospital-acquired infection: prevalence and outcome in infants undergoing open heart surgery in the present era. Indian J Crit Care Med.

[17] Poncelet A.J., Lengele B., Delaere B., Zech F., Glineur D., Funken J.-C. (2008). Algorithm for primary closure in sternal wound infection: a single institution 10-year experience. Eur J Cardio Thorac Surg.

[18] Tadros M.A., Williams V.R., Plourde S., Callery S., Simor A.E., Vearncombe M. (2013). Risk factors for Staphylococcus aureus surgical site infection during an outbreak in patients undergoing cardiovascular surgery. Am J Infect Control.

[19] Wang D., Lu Y., Sun M., Huang X., Du X., Jiao Z. (2022). Pneumonia after cardiovascular surgery: incidence, risk factors and interventions. Front Cardiovasc Med.

[20] Mork C., Gahl B., Eckstein F., Berdajs D.A. (2023). Prolonged cardiopulmonary bypass time as predictive factor for bloodstream infection. Heliyon.

[21] Mureșan A.V., Hălmaciu I., Arbănași E.M., Kaller R., Arbănași E.M., Budișcă O.A. (2022). Prognostic Nutritional Index, Controlling Nutritional Status (CONUT) score, and inflammatory biomarkers as predictors of deep vein thrombosis, acute pulmonary embolism, and mortality in COVID-19 patients. Diagnostics.

[22] Wen B., Lu Y., Huang X., Du X., Sun F., Xie F. (2023). Influence and risk factors of postoperative infection after surgery for ischemic cardiomyopathy. Front Cardiovasc Med.

[23] Lex D.J., Tóth R., Cserép Z., Breuer T., Sápi E., Szatmári A. (2013). Postoperative differences between colonization and infection after pediatric cardiac surgery – a propensity matched analysis. J Cardiothorac Surg.

[24] Aryafar A., Di Marzio A., Guillard O., Pontailler M., Vicca S., Bojan M. (2019). Procalcitonin concentration measured within the first days of cardiac surgery is predictive of postoperative infections in neonates: a case–control study. Pediatr Cardiol.

[25] Li Q., Zheng S., Zhou P.Y., Xiao Z., Wang R., Li J. (2021). The diagnostic accuracy of procalcitonin in infectious patients after cardiac surgery: a systematic review and meta-analysis. J Cardiovasc Med.

[26] Evans L., Rhodes A., Alhazzani W., Antonelli M., Coopersmith C.M., French C. (2021). Surviving sepsis campaign: international guidelines for management of sepsis and septic shock 2021. Intensive Care Med.

[27] Adamik B., Kübler-Kielb J., Golebiowska B., Gamian A., Kübler A. (2000). Effect of sepsis and cardiac surgery with cardiopulmonary bypass on plasma level of nitric oxide metabolites, neopterin, and procalcitonin: correlation with mortality and postoperative complications. Intensive Care Med.

